# Neonatal tetanus with good outcomes at a regional referral hospital in Eastern Uganda: a case report

**DOI:** 10.1186/s13256-022-03255-4

**Published:** 2022-01-31

**Authors:** Clare Nakubulwa, Emmanuel Opio, Gladys Sarah Alekat, Medrine Kibetenga, Florence Olwedo Egwau Alaroker

**Affiliations:** 1 Department of Paediatrics and Child Health, Soroti Regional Referral Hospital, P.O Box 289, Soroti, Uganda; 2 Soroti Regional Referral Hospital, Soroti, Uganda; 3 Nursing Department, Soroti Regional Referral Hospital, Soroti, Uganda

**Keywords:** Neonatal tetanus, Low-income setting, Neonate

## Abstract

**Background:**

Neonatal tetanus, though now rare in developed countries, is still a significant cause of mortality in developing countries. Mortality, which can be as high as 100% without medical intervention, can be reduced to less than 10% with intensive care. Low-resource settings still lack sophisticated intensive care that has been shown to improve outcomes in high-income countries. However, there are low-cost interventions that have been shown to improve outcomes such as the use of magnesium sulfate in the management of severe tetanus.

**Case presentation:**

A 9-day-old term Itesot neonate presented to our facility with inability to breast feed, excessive crying, and stiffening of the body when touched that started on the fourth day of life. On admission, she had signs of respiratory distress, fever, and labile heart rate. A diagnosis of neonatal tetanus with autonomic dysfunction was made, and the neonate was started on diazepam and magnesium sulfate infusion. She showed remarkable improvement and was discharged after 24 days of inpatient care.

**Conclusion:**

There is still need to improve case management modalities for neonatal tetanus in low-income settings to improve outcomes. This case report summarizes how adopting a low-cost treatment modality for neonatal tetanus resulted in good outcomes at a regional referral hospital in Eastern Uganda.

## Introduction

Neonatal tetanus, though preventable by maternal vaccination and aseptic delivery as well as cord care, is still a common cause of morbidity and mortality in developing countries [1–3]. It is caused by a toxin of *Clostridium tetani*, a Gram-positive spore-forming anaerobe and its incubation period ranges from 3 to 10 days. A confirmed case of neonatal tetanus is defined as a neonate with history of all three of the following: (A) normal feeding and crying over the first 2 days of life; (B) onset of illness between 3 and 28 days of life; and (C) inability to suckle (trismus), followed by stiffening (generalized muscle rigidity) and/or convulsions (muscle spasms) [1].

The diagnosis is clinical and therefore requires a high index of suspicion and knowledge on how tetanus presents by the clinician. Although neonatal meningitis or sepsis may present with some features similar to neonatal tetanus, there is no lock jaw in neonatal meningitis and consciousness is spared in neonatal tetanus [1].

Case management involves: eradication of *C. tetani*, neutralizing the circulating toxins, control of spasms, supportive care including oxygen support, fluid management, nutrition, minimal stimulation, and prevention of recurrence through vaccination.

Although the incidence of neonatal tetanus has decreased over the years with the advent of mass vaccination of women of child-bearing age, the case fatality rate remains high [1, 2, 4]. This is especially true for resource-limited settings without modern intensive care, where mortality can be as high as 80% [3, 5, 6]. This mortality is attributed to autonomic nervous system dysfunction (labile hypertension and unstable heart rate) and spasm of respiratory muscles leading to respiratory failure. There have also been reports of residual brain damage from survivors of neonatal tetanus [4].

This case report summarizes good outcomes of neonatal tetanus thought to have resulted from insufficient maternal tetanus toxoid vaccination during pregnancy and environmental exposure to *C. tetani*.

## Case summary

A full-term Itesot female neonate (birth weight 2800 g) delivered by spontaneous vaginal delivery from a health center III was referred to Soroti Regional Referral Hospital special Care Unit (SCU) at 9 days of life. She cried immediately after delivery with a recorded Apgar score of 8 at 1 minute and 9 at 5 minutes. The neonate was stable, breastfed well, and was discharged the next day in a good condition. On the fourth day of life, the mother reported abnormal behavior that was of gradual onset. She noticed excessive crying, tightness of the baby’s lips with progressive difficulty in breast feeding, and stiffening of the body especially when touched. By the ninth day of life, the baby could no longer breast feed and developed a fever that prompted the parents to seek care at a nearby health facility. They were then referred to our facility with a diagnosis of neonatal sepsis.

The mother reported having received only one injection of the tetanus toxoid during the third trimester of pregnancy and did not remember receiving similar vaccines in her childhood or teenage age. She also reported that the floor of their house had just been freshly smeared with cow dung and the neonate had been sleeping on a thin mattress laid directly on the floor. She, however, reported no application of cow dung or other substances on the baby’s umbilical cord.

On admission, we found the neonate febrile at 39 °C, fully conscious but with lockjaw and facial and generalized rigidity. She also demonstrated increased abdominal muscle rigidity and generalized spasms when touched. The umbilical stamp, though still wet, was clean with no sign of infection. She had tachycardia at 172 beats per minute and tachypnea at 72 breaths per minute, with bronchovesicular breath sounds and transmitted sounds but low oxygen saturation ranging from 76% to 82% on room air. A diagnosis of neonatal tetanus with autonomic dysfunction was made at this point with a differential of neonatal meningitis.

**Fig. 1:**
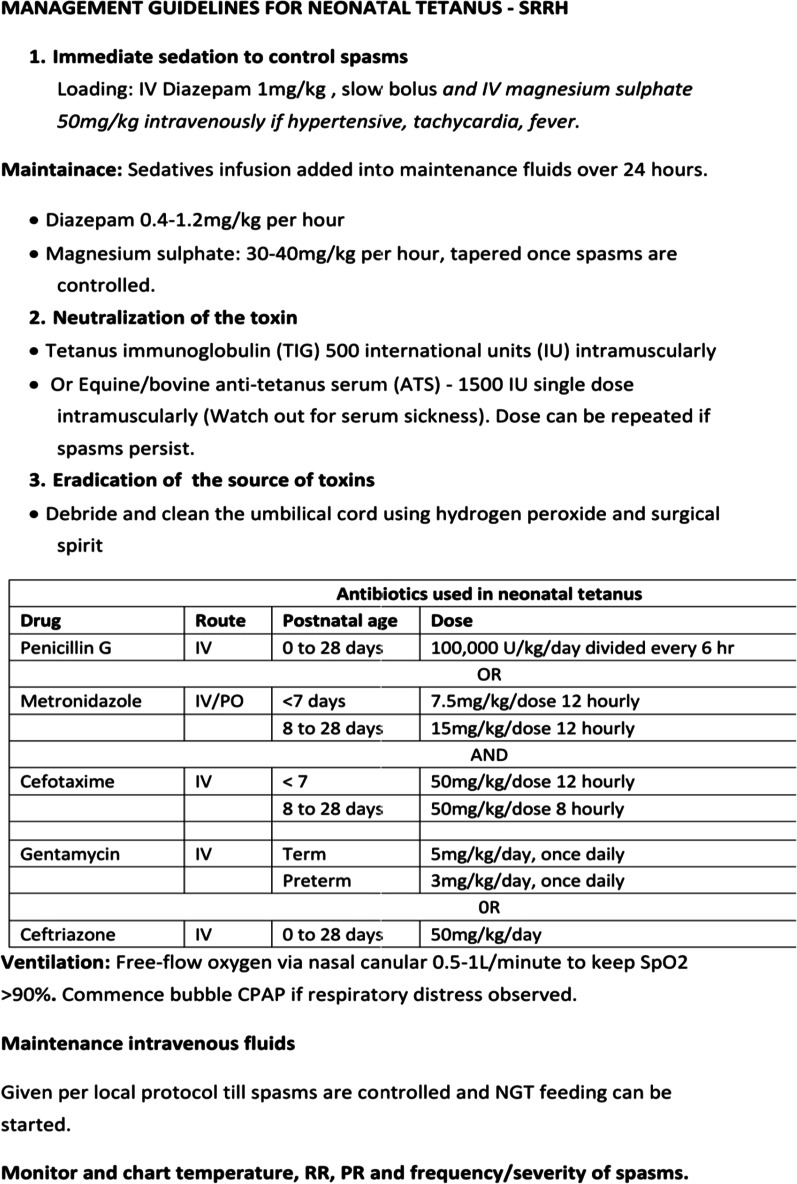
Neonatal tetanus treatment protocol used at Soroti Regional Referral Hospital

**Fig. 2:**
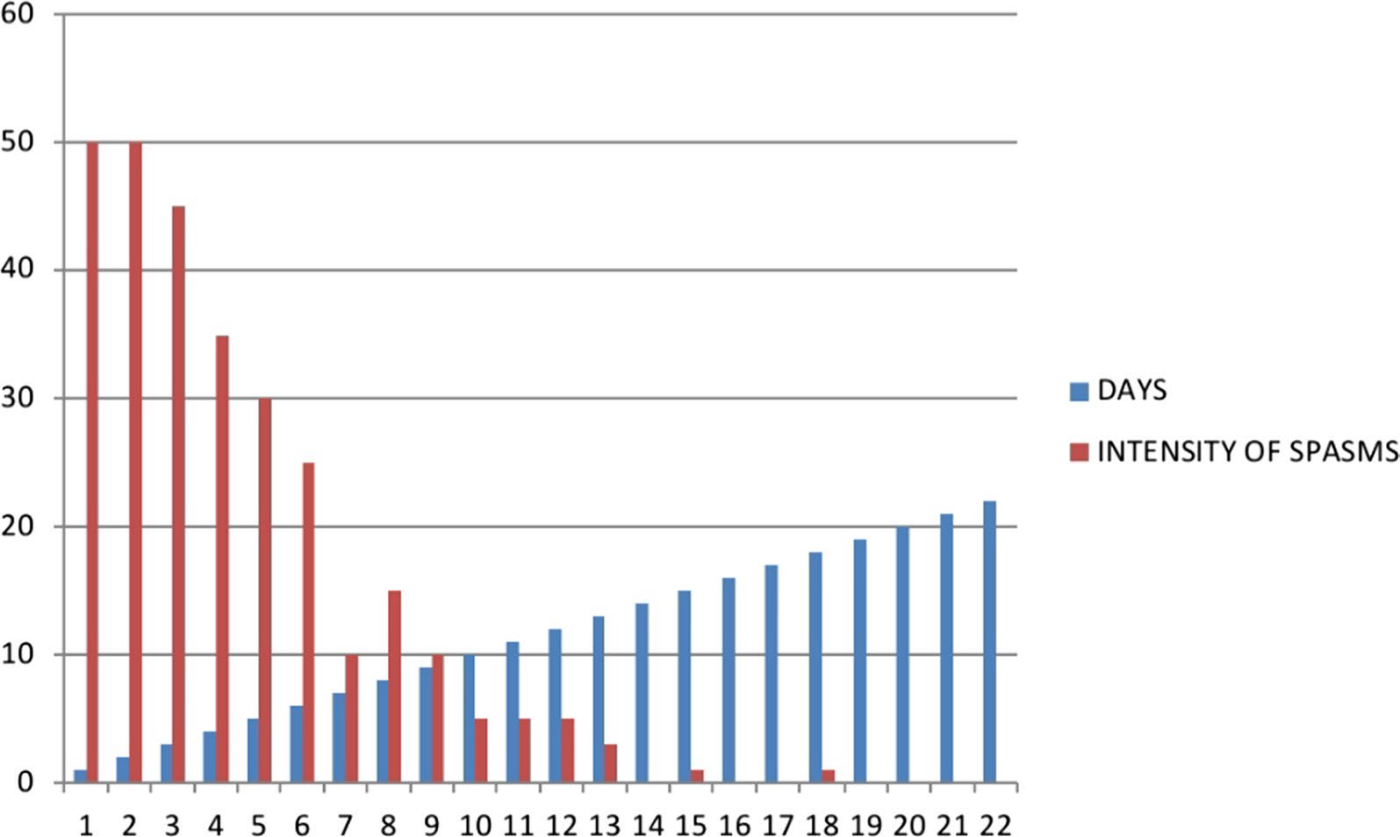
Frequency of spasms in the neonate managed for generalized tetanus

The complete blood count done showed a normal hemogram for her age, and a blood smear revealed no malaria parasites. Biochemical results also revealed normal renal function as well as serum electrolytes but with a slight elevation in serum bilirubin levels. Given the unstable clinical state, a lumbar puncture for cerebral spinal fluid (CSF) analysis was not done and neither was blood culture taken (services not available at the facility). She was started on:

1. Supportive care: oxygen therapy at 0.5 L/minute by nasal prongs, nursed in a dark room and given maintenance intravenous (IV) fluids with dextrose 10% and Ringer’s lactate. Nasogastric tube insertion was attempted on admission but was unsuccessful due to very frequent spasms.
2. IV antibiotics; cefotaxime 50 mg/kg per dose eight-hourly, gentamycin at 5 mg/kg once daily, and metronidazole 7.5 mg/kg per dose 12-hourly for 14 days.
3. Spasm control: IV diazepam loaded at 1 mg/kg, then maintained at 0.3 mg/kg/hour. IV magnesium sulfate loading at 50 mg/kg, then maintenance of 30 mg/kg/hour as per local protocol (Fig. 1). We monitored the frequency and intensity of spasms (Fig. 2). There was no improvement in spasms after 24 hours, so we increased the diazepam infusion to 0.4 mg/kg/hour.
4. With no access to tetanus immunoglobulin, we gave her two doses of intramuscular (IM) equine tetanus antitoxin (TAT) serum, each containing 1500 IU on days 2 and 3 of admission.

Once the nasal gastric tube insertion was successful, we initiated expressed breast milk for nutrition.

Physiotherapy was initiated on the 19th day of admission when spasms had stopped; the nasogastric tube was removed, and breast feeding was restarted.

She was discharged after 24 days of in-patient care, in a stable state on oral diazepam at 0.3 mg/kg/hour given six-hourly. On review 2 weeks later, she was well, with normal tone and reflexes, and was breast feeding well with no reoccurrence of spasms. Oral diazepam was tapered off successfully, and the infant is now growing well with no neurologic sequelae. She was immunized as per the immunization schedule for infants, and the mother got two doses of tetanus toxoid vaccine after discharge as per World Health Organization (WHO) recommendation.

## Discussion

Low-resource settings continue to report high mortality from neonatal tetanus, due to lack of sophisticated management modalities like neuromuscular blockade and invasive ventilation [1–3, 6]. This high mortality has especially been reported in those with symptom onset within the first week of life [3, 7], and in our case symptoms started on the fourth day of life. Earlier presentation with disease may be due to higher inocula of *C. tetani* bacteria, and/or younger infants may be physiologically at higher risk of death [4].

Our patient presented with features of autonomic dysfunction (labile heart rate, tachypnea, and hyperpyrexia) and respiratory distress, which has been associated with high mortality in cases of neonatal tetanus [4, 6, 7]. In well-resourced settings, when benzodiazepines alone fail to control spasms, neuromuscular blockage and ventilation are instituted with good outcomes [8]. These modalities control respiratory muscle spasms and reduce the risk of death from respiratory muscle failure, a major cause of death in neonatal tetanus. However, in our setting that has no access to mechanical ventilation, we adopted the use of magnesium sulfate alongside diazepam and achieved a good outcome.

Magnesium sulfate causes muscle relaxation, vasodilation, and lowering of heart rate, which can help mitigate autonomic dysfunction [9] and has been associated with good outcomes in places with limited intensive care [6, 10].

Other case management components include eradication of *C. tetani* using broad-spectrum antibiotics together with umbilical cord debridement and cleaning with hydrogen peroxide and surgical spirit. Neutralization of circulating tetanus toxin is best done with intramuscular tetanus immunoglobulin (TIG), but in our case we used two doses of TAT because we had no access to TIG. TAT has been suggested as an alternative to the tetanus immunoglobulin, but care should be taken during administration given the risk of serum sickness. Our patient did not exhibit any reaction to it. Our patient was also provided with supportive care.

Neonatal tetanus is a vaccine-preventable disease, and two or more doses of tetanus toxoid vaccine to the mother have been shown to reduce neonatal tetanus mortality by 94% [1, 4]. The benefit comes from the transplacental transfer of tetanus antibodies from the mother to the fetus.

In our case, the mother had only received one dose of tetanus toxoid during her pregnancy, which made the neonate more vulnerable to neonatal tetanus. Given that the delivery happened at a health facility with a trained health worker, we believe the infant most likely got exposed to *C. tetani* during the process of handling at home. Their home floor had just been freshly smeared with cow dung at the time the baby was taken home, and cow dung is known to harbor *C. tetani* [11].

Since neonatal tetanus infection does not confer permanent immunity to the infant, primary series of tetanus immunization are recommended, and this was started at 6 weeks. The WHO also recommends that mothers of babies managed for neonatal tetanus should be vaccinated with two doses of tetanus toxoid, at least 4 weeks apart [1], and this was done for the mother.

## Conclusion

This case report highlights the importance of adhering to the WHO recommendation of two or more tetanus toxoid vaccines during pregnancy, early identification of neonatal tetanus, and adopting case management modalities shown to improve outcomes in resource-limited settings.

## Data Availability

All data supporting our findings are available from the authors upon request.

## Abbreviations

IV: Intravenous
IM: Intramuscular
NT: Neonatal tetanus
TAT: Tetanus antitoxin
TIG: Tetanus immunoglobulin

## References

[1] World Health Organization. Tetanus vaccines: WHO position paper, February 2017—recommendations. Vaccine. 2018;36(25):1–14. 10.1016/j.vaccine.2017.02.034 28427847

[2] Patel DR, Sindhal HS, Patel DV, Nimbalkar SM. Neonatal tetanus: case series. J Clin Neonatol. 2016;5(2):2015–7.

[3] Okidi R. Neonatal tetanus in St. Mary’s Hospital Lacor: a case report. Clin Case Rep. 2020;8(March):2234–6. 10.1002/ccr3.3091 PMC7669404 33235766

[4] Ibinda F, Bauni E, Kariuki SM, Fegan G, Lewa J. Incidence and risk factors for neonatal tetanus in admissions to Kilifi County. PLoS ONE. 2015;85:1–13. 10.1371/journal.pone.0122606. PMC4388671 25849440

[5] Olum S, Eyul J, Lukwiya DO, Scolding N. Tetanus in a rural low-income intensive care unit setting. Brain Commun. 2021. 10.1093/braincomms/fcab013. PMC8010432 33824951

[6] Burgoine K, Egiru E, Ikiror J, Acom L, Akol S, Olupot-Olupot P. Neonatal tetanus in eastern Uganda: improved outcome following the implementation of a neonatal tetanus protocol. Trop Doct. 2015;50(1):57–62. 10.1177/0049475519872860 31474192

[7] Khanh P, Trieu HT, Nadia I, Lubis D, Loan HT, Thi T, *et al*. Prognosis of neonatal tetanus in the modern management era: an observational study in 107 Vietnamese infants. Int J Infect Dis. 2015;33:7–11. 10.1016/j.ijid.2014.12.011. PMC4396701 25499039

[8] Chang S, Wang C. Neonatal tetanus after home delivery: report of one case. Pediatr Neonatol. 2010;51(3):182–5. 10.1016/S1875-9572(10)60034-4. 20675244

[9] Karanikolas M, Velissaris D, Marangos M, Karamouzos V, Fligou F, Filos KS. Prolonged high-dose intravenous magnesium therapy for severe tetanus in the intensive care unit: a case series. J Med Case Rep. 2010;4(100):2–6. 10.1186/1752-1947-4-100 PMC2862042 20356376

[10] Trieu HT, Lubis IN, Qui PT, Yen LM, Wills B, Thwaites CL, *et al*. Neonatal tetanus in Vietnam: comprehensive intensive care support improves mortality. J Pediatr Infect Dis Soc. 2016;5(2):227–30. 10.1093/jpids/piv059 PMC5407131 26407289

[11] Emeribe VC, Akah LU. Neonatal tetanus in African children: causes, symptoms, predisposing factors, prevention and control. Arts Soc Sci J. 2011;2011:21–4.

